# *Campylobacter concisus* Impairs Sodium Absorption in Colonic Epithelium via ENaC Dysfunction and Claudin-8 Disruption

**DOI:** 10.3390/ijms21020373

**Published:** 2020-01-07

**Authors:** Praveen Kumar Nattramilarasu, Roland Bücker, Fábia Daniela Lobo de Sá, Anja Fromm, Oliver Nagel, In-Fah Maria Lee, Eduard Butkevych, Soraya Mousavi, Claudia Genger, Sigri Kløve, Markus M. Heimesaat, Stefan Bereswill, Michal R. Schweiger, Hans Linde Nielsen, Hanno Troeger, Jörg-Dieter Schulzke

**Affiliations:** 1 Institute of Clinical Physiology/Nutritional Medicine, Medical Department, Division of Gastroenterology, Infectiology and Rheumatology, Charité—Universitätsmedizin Berlin, 12203 Berlin, Germany; 2 Institute of Microbiology, Infectious Diseases and Immunology, Charité—Universitätsmedizin Berlin, Campus Benjamin Franklin, 14195 Berlin, Germany; 3 Laboratory for Epigenetics and Tumour genetics, University Hospital Cologne and Centre for Molecular Medicine Cologne, 50931 Cologne, Germany; 4 Department of Clinical Microbiology, Aalborg University Hospital, 9000 Aalborg, Denmark; 5 Department of Clinical Medicine, Aalborg University, 9000 Aalborg, Denmark; 6 Medical Department, Division of Gastroenterology, Infectiology and Rheumatology, Charité—Universitätsmedizin Berlin, 12203 Berlin, Germany

**Keywords:** sodium transport, epithelial sodium channel, extracellular signal-regulated kinase, *Campylobacter concisus*, diarrhea, tight junction, claudin-8

## Abstract

The epithelial sodium channel (ENaC) can increase the colonic absorptive capacity for salt and water. *Campylobacter concisus* is a common pathogenic epsilonproteobacterium, causing enteritis and diarrhea. It can induce barrier dysfunction in the intestine, but its influence on intestinal transport function is still unknown. Therefore, our study aimed to characterize *C. concisus* effects on ENaC using the HT-29/B6-GR/MR (epithelial cell line HT-29/B6 transfected with glucocorticoid and mineralocorticoid receptors) cell model and mouse colon. In Ussing chambers, *C. concisus* infection inhibited ENaC-dependent Na^+^ transport as indicated by a reduction in amiloride-sensitive short circuit current (−55%, *n* = 15, *p* < 0.001). This occurred via down-regulation of β- and γ-ENaC mRNA expression and ENaC ubiquitination due to extracellular signal-regulated kinase (ERK)1/2 activation, predicted by Ingenuity Pathway Analysis (IPA). In parallel, *C. concisus* reduced the expression of the sealing tight junction (TJ) protein claudin-8 and induced claudin-8 redistribution off the TJ domain of the enterocytes, which facilitates the back leakage of Na^+^ ions into the intestinal lumen. In conclusion, *C. concisus* caused ENaC dysfunction via interleukin-32-regulated ERK1/2, as well as claudin-8-dependent barrier dysfunction—both of which contribute to Na^+^ malabsorption and diarrhea.

## 1. Introduction

*Campylobacter concisus* (*C. concisus*) is a Gram-negative, hydrogen (H_2_)-utilizing microorganism, first identified in periodontal pockets [1]. Extensive colonization by *C. concisus* and other anaerobic bacteria contributes to inflammation of the oral mucosa [2,3]. A clinical study first detected *C. concisus*, zoonotic *Campylobacter jejuni*/*Campylobacter coli* and other *Campylobacter* spp. in fecal samples of children with diarrhea, whereas fecal samples of adult patients with diarrhea mainly contained *C. jejuni*/*C. coli* without *C. concisus* [4]. *C. concisus* is also a frequent cause of diarrhea in immunocompromised patients [5]. However, *C. concisus* has been identified in oral and fecal samples of healthy patients with the same frequency as in diarrheal patients [6,7]. Thus, it has been difficult to determine whether *C. concisus* has a role in the pathophysiology of acute infective diarrhea.

A large cohort study revealed that *C. concisus* infection caused watery stools in most patients with diarrhea, which was prolonged compared to patients with diarrhea caused by *C. jejuni* [8]. Furthermore, *C. concisus* promoted intestinal barrier dysfunction [9], although the effects of *C. concisus* on intestinal transport function remains unclear. While sodium-hydrogen exchanger 3 (NHE3)-mediated electroneutral Na^+^ transport predominates in ileum and proximal colon, epithelial sodium channel (ENaC)-mediated electrogenic Na^+^ absorption is more important in distal colon, especially when activated by corticoids in diarrheal states [10]. ENaC is composed of three subunits (α, β and γ) [11]. α-ENaC is constitutively expressed, whereas β- and γ-ENaC expression is regulated by gluco- and mineralocorticoids [12]. Enhanced Na^+^ absorption via activated ENaC in the distal colon is accompanied by transcriptional up-regulation of β- and γ-ENaC-subunits [13,14].

To study ENaC activity in vitro, we used HT-29/B6-GR/MR cell model, in which classical human intestinal epithelial cells HT-29/B6 are stably transfected with glucocorticoid (GR) and mineralocorticoid (MR) receptors. Glucocorticoid receptor (GR)/mineralocorticoid receptor (MR) activation is crucial for ENaC activity in this in vitro model [15]. Furthermore, other regulatory inputs, such as the phosphorylation and activation of mitogen-activated protein kinase (MAPK) p38, extracellular signal-regulated kinase (ERK), c-Jun N-terminal kinase (JNK) and signal transducer and activator of transcription 6 (STAT-6), influence ENaC function [16,17]. Among different MAPK enzymes, ERK activation plays a central role in inhibiting ENaC function in inflammatory conditions such as ulcerative colitis, lymphocytic colitis and Crohn’s disease [18,19,20].

*C. concisus* also induces intestinal epithelial barrier dysfunction through apoptosis and moderate modifications to tight junctions (TJ) [9], which supports the concept of a leak-flux diarrheal pathomechanism. The main aim of this study was to determine whether *C. concisus* impairs ENaC-dependent Na^+^ transport in the colon, which would implicate Na^+^ malabsorption in the pathogenesis of diarrhea caused by *C. concisus*.

TJs are important components of the intestinal epithelial barrier. They seal the paracellular space between enterocytes in the intestinal epithelium (fence function) [21]. Although the primary role of most TJ proteins like occludin, junctional adhesion molecules (JAM) and tricellulin in intestinal epithelia is to seal the paracellular spaces [22], some TJ proteins of the claudin family (e.g., claudin-2 and -15) act as paracellular channels (gate function) for water and ions [23,24]. In the colon, claudin-8 seals the lateral paracellular space and forms a barrier to prevent back leakage of absorbed Na^+^ into the lumen [25]. In a previous study, we found claudin-8 to be down-regulated in parallel with impaired ENaC-mediated electrogenic Na^+^ absorption in human colon during acute *C. jejuni* infection [26]. However, it remains unclear whether *C. concisus* might also promote down-regulation of claudin-8, which could contribute to diarrhea. Therefore, in addition to an electrophysiological approach to determine the regulatory effects of *C. concisus* on ENaC function, we investigated at a molecular level the effects of *C. concisus* on paracellular barrier disruption, particularly claudin-8 down-regulation, in colonic epithelial cells.

## 2. Results

### 2.1. Campylobacter concisus Impairs Sodium Absorption via ENaC Dysfunction In Vitro

**Figure 1:**
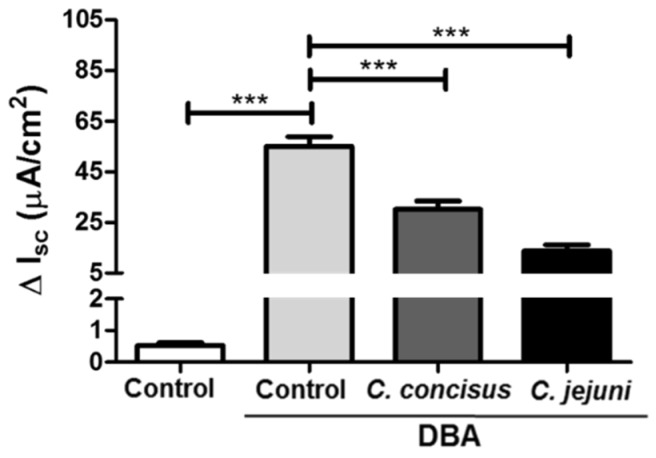
Epithelial sodium channel (ENaC) impairment in HT-29/B6-GR/MR (epithelial cell line HT-29/B6 transfected with glucocorticoid and mineralocorticoid receptors) cells 48 h after *Campylobacter concisus* and *Campylobacter jejuni* infections. Changes in short circuit current (ΔI_SC_ in µA/cm^2^) were recorded in Ussing chambers followed by 100 µM amiloride addition to the apical compartment of the Ussing chamber (*n* = 15, *** *p* < 0.001). HT-29/B6-GR/MR cells were stimulated with DBA from both apical and basolateral sides of the cell monolayers. DBA; glucocorticoid dexamethasone (D, 50 nM), butyrate (B, 2 mM sodium salt) and mineralocorticoid aldosterone (A, 3 nM).

ENaC-dependent Na^+^ absorption in vitro was studied using HT-29/B6-GR/MR colonic cell monolayers. We then established a model of infected HT-29/B6-GR/MR (epithelial cell line HT-29/B6 transfected with glucocorticoid receptors (GR) and mineralocorticoid receptors (MR); [15]) cells to study the effects of *C. concisus* on ENaC-dependent Na^+^ absorption (see also Methods, Section 4.1). Polarized and confluent cell monolayers were treated with dexamethasone, butyrate and aldosterone (DBA) to induce glucocorticoid (GR) and mineralocorticoid (MR) receptors as a means of activating ENaC-dependent Na^+^ absorption prior to infection. An increase in amiloride-sensitive short circuit current (I_SC_ in µA/cm^2^) was observed after DBA stimulation compared with unstimulated controls and recorded as ΔI_SC_ (Figure 1). Forty-eight hours post-infection, a significant reduction in ΔI_SC_ was observed in *C. concisus*-infected cell monolayers, which was similar to that seen with *C. jejuni* infection (Figure 1).

**Figure 2:**
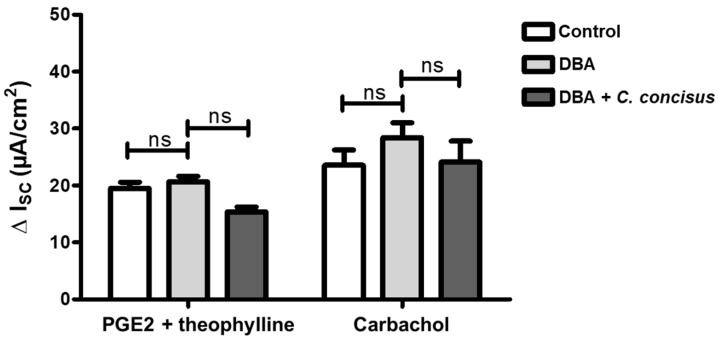
Functional viability of HT-29/B6-GR/MR cell monolayers 48 h after *Campylobacter concisus* infection as indicated by chloride (Cl^−^) channel activation. Cl^−^ secretion was determined as peak increase in short circuit current (ΔI_SC_ in µA/cm^2^) 2–3 min after addition of Prostaglandin E2 (PGE2) (10 µM, basolateral side) and theophylline (10 mM, apical and basolateral side) to the Ussing chamber. Peak ΔI_SC_ was also measured 2–3 min after addition of carbachol (100 µM, basolateral side). *C. concisus*-infected cell monolayers were compared to untreated and DBA-stimulated controls (*n* = 4–5 each, ns = not significant). DBA = dexamethasone, butyrate, and aldosterone.

To confirm that HT-29/B6-GR/MR cells retained functional viability at this time point, electrogenic chloride (Cl^−^) secretion was determined after the addition of prostaglandin E_2_ (PGE2) and theophylline (both acting via cyclic adenosine monophosphate (cAMP) stimulation), or the cholinergic agonist carbachol (acting via calcium as second messenger). No significant difference in the increase in I_SC_ was observed between controls and *C. concisus*-infected cell monolayers, either in response to PGE2 and theophylline, or to carbachol treatment (Figure 2). This confirmed that cells 48 h post-infection were as functionally viable as control cells.

### 2.2. Campylobacter concisus Down-Regulates the mRNA Expression of β- and γ-ENaC Subunits

**Figure 3:**
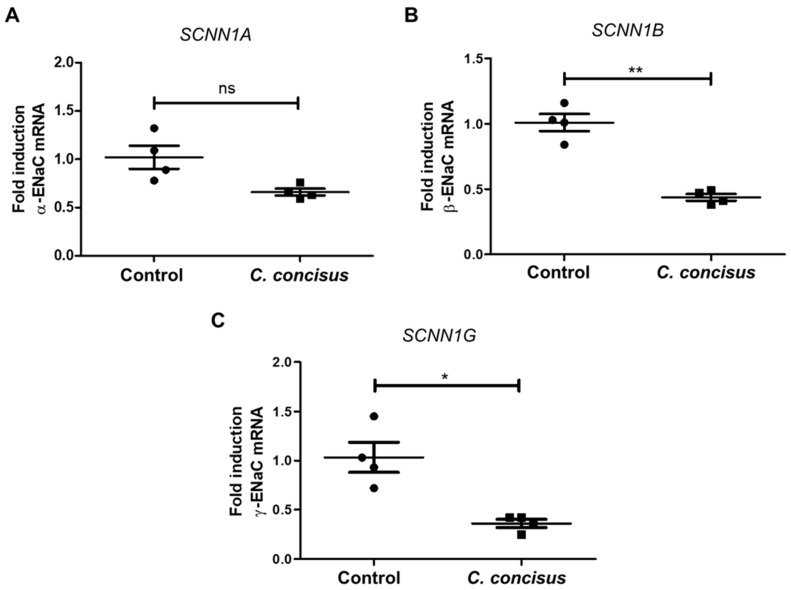
mRNA expression changes in ENaC subunits (-α, -β, -γ) in HT-29/B6-GR/MR cells 48 h after *Camplyobacter concisus* infection through RT-qPCR. (**A**) α-ENaC gene (*SCNN1A*) mRNA expression in DBA-stimulated controls and *C. concisus*-infected cell monolayers (*n* = 4 each, ns = not significant, *p* > 0.05). (**B**) β-ENaC gene (*SCNN1B*) mRNA expression in DBA-stimulated controls and *C. concisus*-infected cell monolayers (*n* = 4 each, ** *p* < 0.01). (**C**) γ-ENaC gene (*SCNN1G*) mRNA expression in DBA-stimulated controls and *C. concisus*-infected cell monolayers (*n* = 4 each, * *p* < 0.05). Changes in ENaC subunit mRNA expression in DBA-stimulated controls with respect to unstimulated controls are indicated in Supplementary Figure S1. DBA = dexamethasone, butyrate, and aldosterone.

*C. concisus* was associated with a significant decrease in the mRNA expression of β- and γ-ENaC subunits compared with DBA-stimulated controls, whereas α-ENaC subunit (*SCNN1A*) mRNA expression was not significantly changed (Figure 3). Thus, *C. concisus* down-regulated the mRNA expression of β- and γ-ENaC genes (*SCNN1B*, *SCNN1G*) might be sufficient to impair ENaC-mediated Na^+^ absorption.

In addition, we determined the mRNA expression of ENaC subunits (-α, -β, -γ) in unstimulated controls, as well as in DBA-stimulated controls and *C. concisus*-infected cells. α-ENaC subunit (*SCNN1A*) mRNA expression was not significantly altered after DBA-stimulation (Supplementary Figure S1A), whereas β- and γ-ENaC (*SCNN1B*, *SCNN1G*) mRNA expressions were significantly increased with DBA stimulation (Supplementary Figure S1B,C).

**Figure 4:**
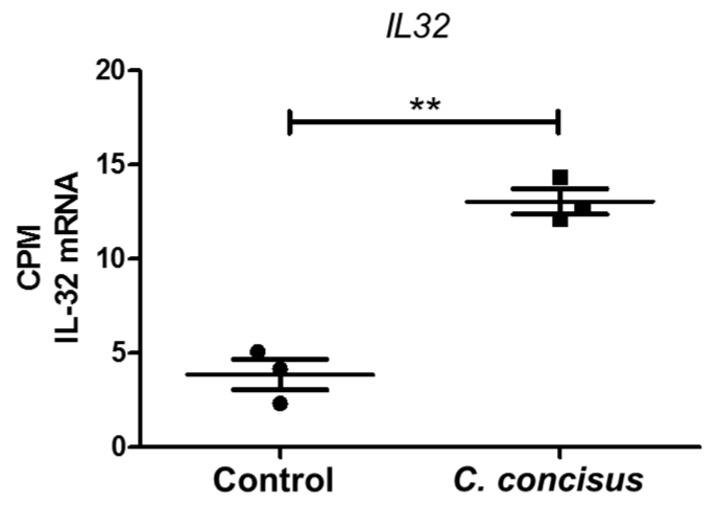
mRNA expression changes in interleukin-32 in HT-29/B6-GR/MR cells 48 h after *Campylobacter concisus* infection. The mRNA expression of the IL-32 gene (*IL32*) in DBA-stimulated controls and *C. concisus*-infected cell monolayers expressed in counts per million (CPM) calculated by differential gene expression analysis using RNA-Seq (*n* = 3, ** *p* < 0.01). DBA = dexamethasone, butyrate, and aldosterone.

Gene expression analysis of RNA-Seq data revealed that 1667 genes were affected (*p* < 0.05) 48 h after *C. concisus* infection. RNA-Seq data are publicly available at Gene Expression Omnibus (GEO) archive under National Centre for Biotechnology Information (NCBI) website with GEO accession ID 141217 [*Campylobacter concisus* impairs sodium absorption via ENaC dysfunction and claudin-8 disruption. Available online: https://www.ncbi.nlm.nih.gov/gds/?term=GSE141217 (1 January 2020)]. The *p*-values, adjusted for multiple testing using the Benjamini–Hochberg procedure, revealed that 186 genes were differentially expressed (adjusted *p* < 0.05)—of which, 66 genes were up-regulated, and 120 genes were down-regulated (Supplementary Table S1). Importantly, the mRNA expression of the pro-inflammatory cytokine interleukin-32 (IL-32) was increased in HT-29/B6-GR/MR cell monolayers 48 h after *C. concisus* infection (Figure 4). Furthermore, the downstream signaling pathways and upstream regulators modulating ENaC function was evaluated by bioinformatics prediction using Ingenuity Pathway Analysis (IPA) software (Supplementary Table S2).

Furthermore, we analyzed the mRNA expression of different absorptive and secretory transporters that influence the Na^+^ absorption and ENaC function after *C. concisus* infection through RNA-seq data (Supplementary Table S2). It revealed that the mRNA expression of Na^+^K^+^ATPase was not down-regulated after *C. concisus* infection. The mRNA expression of secretory chloride channels Na-K-Cl cotransporter 1 (NKCC1) and calcium-activated chloride channels (CaCC) was not up-regulated (as would have been expected for a diarrheal state) after *C. concisus* infection. Cystic fibrosis transmembrane conductance regulator (CFTR) does not impact the inhibition of ENaC function by *C. concisus* either, as the mRNA expression of CFTR was not up-regulated but rather down-regulated. The mRNA expression of NHE3 was not changed after *C. concisus* infection, which might imply the unaltered electroneutral NaCl-absorption during *C. concisus* infection.

### 2.3. C. jejuni and C. concisus Dysregulate ENaC Function via ERK Activation

**Figure 5:**
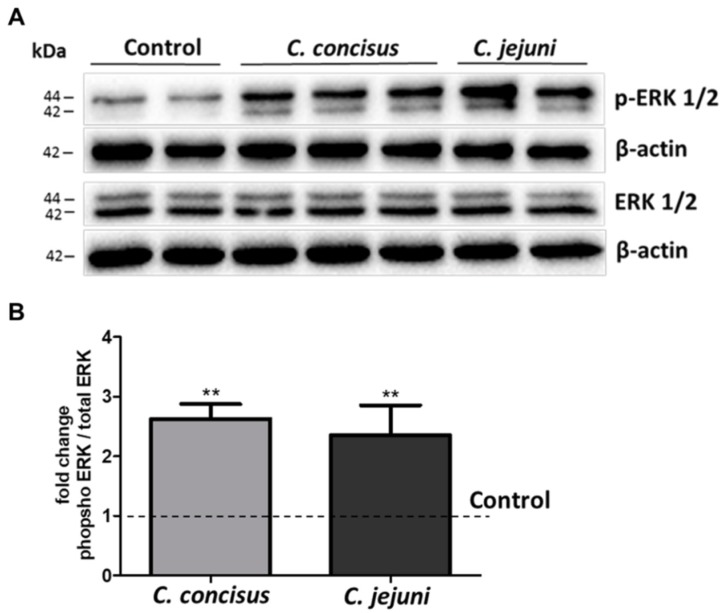
Activation of ERK pathway in HT-29/B6-GR/MR cell monolayers 48 h after *Campylobacter concisus* and *Campylobacter jejuni* infections. (**A**) Western blots of phosphorylated extracellular signal-regulated kinase (ERK) (p-ERK1/2) and total ERK (ERK1/2) 15 min after DBA stimulation and *C. concisus* or *C. jejuni* infection. (**B**) Densitometric analysis of Western blots shown as bar graphs representing fold-change in the band intensity ratio of p-ERK1/2 to ERK1/2 (normalized to β-actin) in *C. concisus* and *C. jejuni* infections compared with DBA-stimulated controls, indicated by dotted line *(n* = 4–6 each, ** *p* < 0.01). DBA = dexamethasone, butyrate, and aldosterone.

Forty-eight hours post-infection, Western blots of phosphorylated ERK (p-ERK1/2) and total ERK (ERK1/2) 15 min after DBA stimulation and *Campylobacter* spp. infection were performed. Phosphorylation of isoform ERK1 (44 kDa band) and ERK2 (42 kDa band) were increased by both *C. concisus* and *C. jejuni* (Figure 5A).

Densitometry analysis revealed that *C. concisus* and *C. jejuni* increased ERK phosphorylation after DBA stimulation (Figure 5B), indicating that *C. concisus* and *C. jejuni* induced ERK activation in parallel with ENaC dysfunction in HT-29/B6-GR/MR cells.

**Figure 6:**
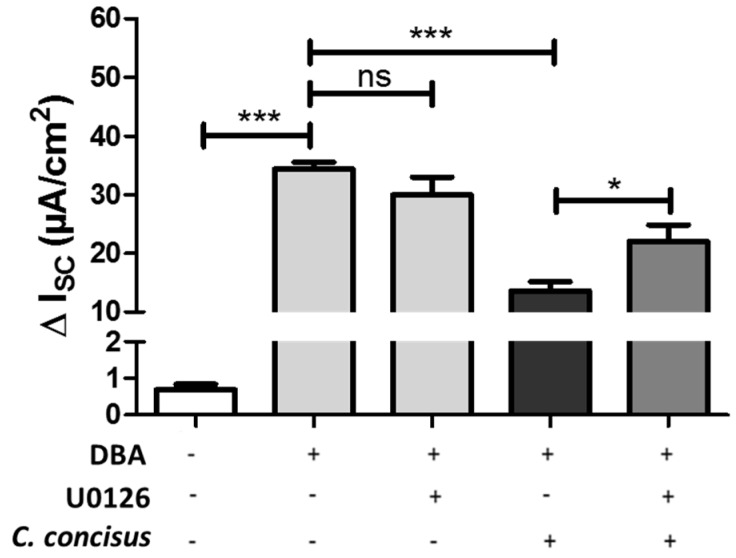
ERK inhibition by U0126 ameliorates the functional impairment of ENaC by *Campylobacter concisus* in HT-29/B6-GR/MR cell monolayers. The specific ERK inhibitor U0126 (10 µM) was applied to the cell monolayers 2 h before *C. concisus* infection. Parallel control monolayers were only stimulated by DBA (dexamethasone, butyrate, and aldosterone) without infection. Amiloride-sensitive short circuit current (I_SC_) was recorded 48 h post-infection to determine ENaC function. The decrease in short circuit current was measured 20 min after addition of amiloride (100 µM) to the apical side (*n* = 6–8, * *p* < 0.05, *** *p* < 0.001, ns = not significant).

To determine whether *C. concisus*-induced ERK activation caused functional impairment of ENaC, the specific inhibitor U0126 was used to block ERK activation by upstream inhibition of MEK. *C. concisus*-induced ENaC dysfunction was then tested again during inhibition of ERK activation. Based on measurements of the amiloride-sensitive increase in I_SC_ 48 h post-infection, U0126 significantly decreased the damaging effect of *C. concisus* infection on ENaC (Figure 6), suggesting that ERK blockade attenuates *C. concisus*-induced ENaC dysfunction.

### 2.4. Campylobacter concisus Decreases Transepithelial Electrical Resistance and Promotes Changes in Tight Junction Protein Expression

**Figure 7:**
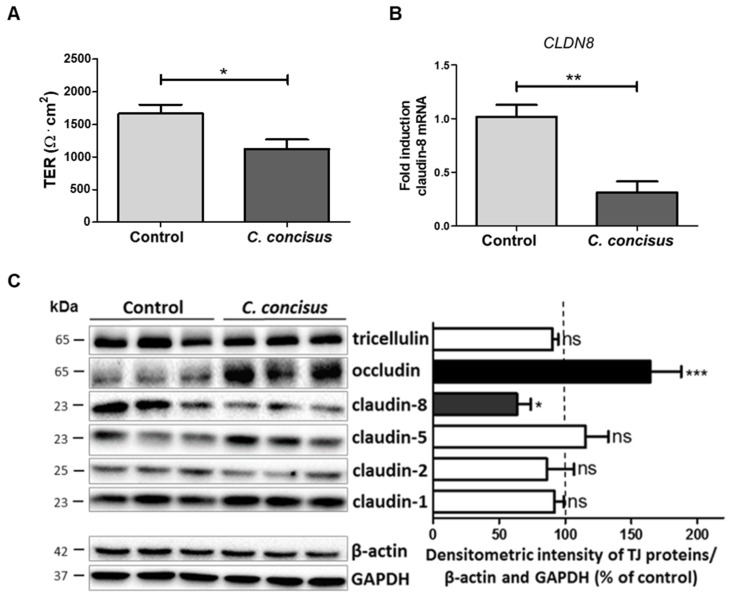
Changes in TER and tight junction protein expression changes in HT-29/B6-GR/MR cell monolayers 48 h after *Campylobacter concisus* infection. (**A**) Transepithelial resistance (TER) was measured 20 min after addition of amiloride (100 µM). *C. concisus*-infected cell monolayers were compared with DBA-stimulated controls (*n* = 7–8, * *p* < 0.05). (**B**) Claudin-8 (*CLDN8*) mRNA expression in *C. concisus*-infected cell monolayers compared with DBA-stimulated controls (*n* = 4, ** *p* < 0.01). (**C**) Western blots and the corresponding densitometric analysis were performed to detect changes in tight junction protein expression after *C. concisus* infection compared with controls after DBA stimulation (*n* = 3–5, * *p* < 0.05, *** *p* < 0.001, ns = not significant). DBA = dexamethasone, butyrate, and aldosterone.

To investigate the barrier function of infected cell monolayers 48 h post-infection, transepithelial electrical resistance (TER) was measured 20 min after adding amiloride, when ENaC was completely blocked. Under these conditions, TER reflected paracellular sealing by TJ proteins. In *C. concisus*-infected cell monolayers, TER 48 h post-infection was decreased compared with DBA-stimulated controls (Figure 7A). This is direct evidence that *C. concisus* impaired paracellular barrier function. We also examined changes in TJ integrity at the molecular level, and using RT-qPCR, found claudin-8 (*CLDN8*) mRNA expression to be decreased (Figure 7B).

We also analyzed the expression of different TJ proteins by Western blotting and densitometric analysis, which indicated that *C. concisus* decreased claudin-8 expression and increased occludin expression when compared with DBA-stimulated controls (Figure 7C). The expression of the other TJ proteins was not affected by *C. concisus* infection.

**Figure 8:**
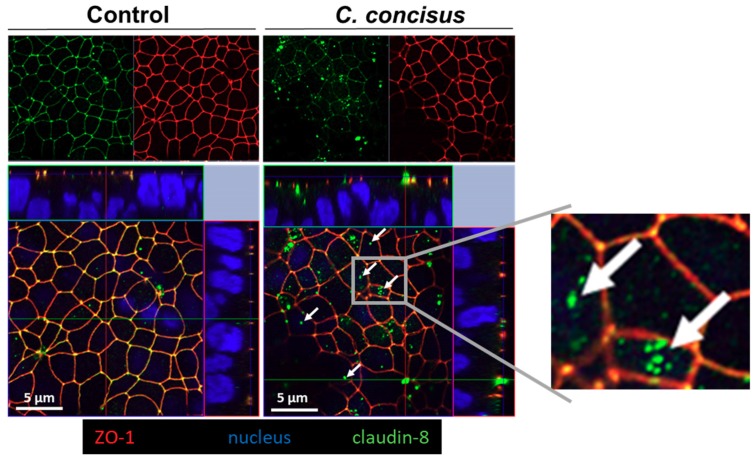
Subcellular redistribution of claudin-8 in *Campylobacter concisus*-infected HT-29/B6-GR/MR cell monolayers 48 h post-infection. Claudin-8 (green) and zonula occludens protein-1 (ZO-1) (red) co-localized in the tight junction of DBA-stimulated control monolayers. Nuclei (blue) were stained by 4′-6-diamidino-2-phenylindole dihydrochloride (DAPI). In *C. concisus*-infected cell monolayers, claudin-8 was redistributed from continuous tight junction strands into intracellular compartments, indicated by white arrows. DBA = dexamethasone, butyrate, and aldosterone.

In order to further study the functional importance of this change in claudin-8, parallel experiments were done to determine the effect of *C. concisus* infection on the subcellular distribution of claudin-8 using confocal laser-scanning microscopy (CLSM). We observed subcellular redistribution of claudin-8 protein signals away from the TJ. Z-stack analysis of CLSM images revealed that claudin-8 was delocalized from TJs and accumulated as intracellular aggregates in *C. concisus*-infected cell monolayers, whereas clear co-localization of zonula occludens protein-1 (ZO-1) and claudin-8 was observed in TJs in control cell monolayers (Figure 8).

**Figure 9:**
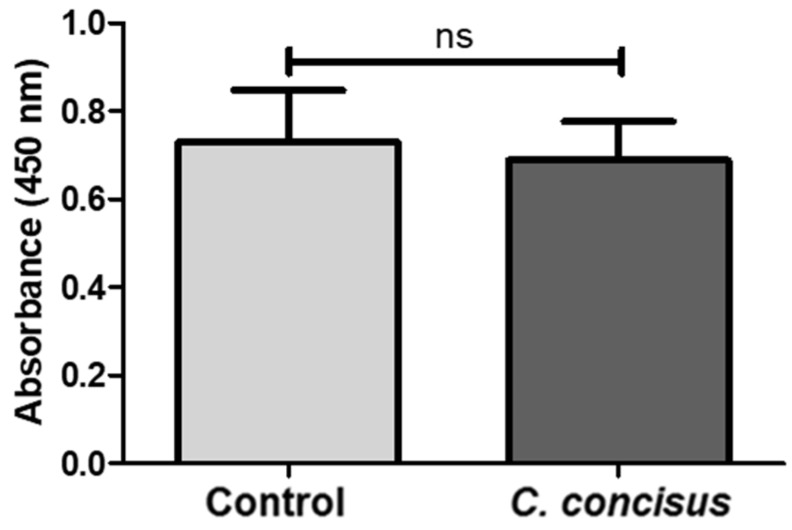
Evaluation of cell viability in *Campylobacter concisus*-infected HT-29/B6-GR/MR cell monolayers 48 h post-infection. Absorbance values were recorded at 450 nm with a reference wavelength of 600 nm in DBA-stimulated control cells and *C. concisus*-infected cells, which reflected cellular activity 2 h after addition of water-soluble tetrazolium salt (WST-8 in the CCK8 assay; *n* = 8, ns *p* > 0.05, ns = not significant). DBA = dexamethasone, butyrate, and aldosterone.

To confirm cell viability 48 h after *C. concisus* infection, cell proliferation rate and cytotoxicity of the cells were tested using the CCK-8 (Cell Counting Kit-8) assay. This revealed no significant differences in the cell viability after *C. concisus* infection when compared with controls (Figure 9), indicating that *C. concisus*-induced paracellular barrier defects were independent of cytotoxicity.

### 2.5. Campylobacter concisus Impairs Sodium Absorption via ENaC in the Colon of IL-10^−/−^ Mouse

**Figure 10:**
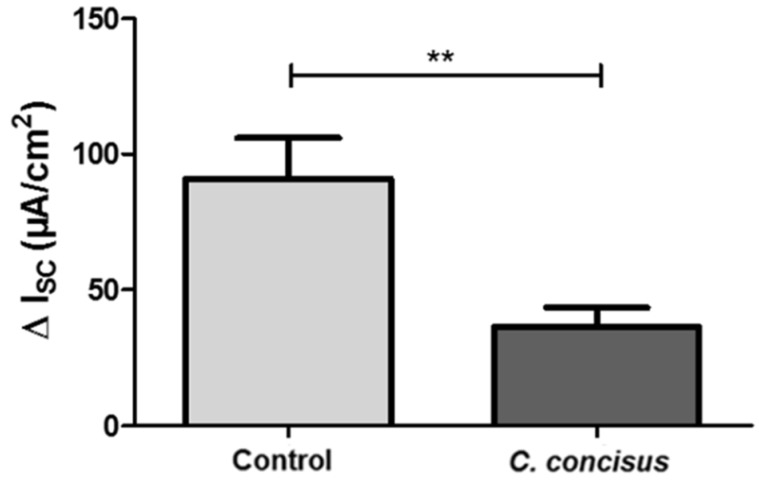
Impaired ENaC function in colon of *Campylobacter concisus*-infected abiotic IL-10^−/−^ mice 6 days post-infection. Colon specimens were obtained from infected mice and stimulated with the mineralocorticoid aldosterone (3 nM) in the Ussing chamber for 6 h. The colon samples of IL-10^−/−^ mice infected with commensal *Escherichia coli* were used as controls. The decrease in short circuit current (ΔI_SC_; µA/cm^2^) 20 min after addition of amiloride (100 µM) was measured and represented ENaC-dependent Na^+^ absorption (*n* = 8–9, ** *p* < 0.05).

The abiotic IL-10^−/−^ mouse is an ideal model to study the functionality of inflamed intestine following experimental *Campylobacter jejuni* infection [27,28]. This mouse model was used to determine the transport effects of *C. concisus* in vivo, particularly ENaC-mediated Na^+^ absorption in infected distal colon. Similar to our in vitro cell monolayer model, changes in Isc across tissues obtained from infected IL-10^−/−^ mice were measured in Ussing chambers. Six days post-infection, *C. concisus* infection in IL-10^−/−^ mouse colon caused a decrease in the amiloride-sensitive I_SC_ when compared with control mice, indicating marked ENaC dysfunction (Figure 10).

In order to test the viability of colonic tissue in Ussing chambers 6 h after aldosterone stimulation, we determined changes in I_SC_ (∆I_SC_, µA/cm^2^) after stimulation of electrogenic Cl^−^ secretion by prostaglandin E_2_ (PGE2) and theophylline, and its subsequent inhibition by bumetanide. As shown in Table 1, in all mucosae Cl^−^ secretion was stimulated by PGE2 and theophylline and inhibited by bumetanide. No significant differences in ∆I_SC_ were observed between controls and *C. concisus*-infected IL-10^−/−^ mice, indicating that mucosal viability was maintained (Table 1). Furthermore, impedance measurements indicated that there were no differences in epithelial resistance (R^epi^) and subepithelial resistance (R^sub^) between the two groups (Table 1), which implies that colonic ENaC dysfunction induced by *C. concisus* in IL-10^−/−^ mice was independent of epithelial barrier changes (e.g., leaks or tissue destruction).

## 3. Discussion

The first main finding was that *C. concisus* infection impaired ENaC activity in colonic epithelial cells, which was reflected by a decrease in amiloride-sensitive I_SC_ and the transcriptional down-regulation of β- and γ-ENaC subunits in our HT-29/B6-GR/MR (HT-29/B6 colonic epithelial cells stably transfected with glucocorticoid receptors (GR) and mineralocorticoid receptors (MR)) cell model in vitro. The HT-29/B6-GR/MR epithelial cell model is the only steroid hormone-sensitive intestinal cell model available. Basic glucocorticoids levels like 50 nM dexamethasone in the present study are necessary for a localization of the de novo expressed ENaC subunits in the apical enterocyte cell membrane. Butyrate inhibits histone deacetylation and thereby intensify β- and γ-ENaC subunit expression via increased binding of the transcription factor SP3 and histone acetylation [29]. Put together, the HT-29/B6-GR/MR epithelial cell model also allowed us to investigate the intracellular cell signaling pathways that regulate or impair ENaC function.

During diarrheal states, ENaC-mediated electrogenic sodium absorption is activated in the distal colon as a reserve absorption system to minimize the loss of Na^+^. *C. concisus* impaired ENaC-mediated Na^+^ absorption in this cell model. ENaC dysfunction has been identified as a pathomechanism that reduces the overall transport capacity for Na^+^ and directly contributes to Na^+^ malabsorption and watery diarrhea, a predominant intestinal symptom in *C. concisus* infection [8]. Furthermore, *C. concisus* was frequently detected in fecal samples of diarrheal patients [4,5]. Interestingly, *C. concisus* is the main non-zoonotic *Campylobacter* species identified so far in human specimens and a source of infection is yet to be identified in the environment or animals. Indeed, for many years it was unclear whether colonization of *C. concisus* in the human intestinal mucosa is cause or consequence of intestinal inflammation. Nielsen and co-workers demonstrated that *C. concisus* induced barrier dysfunction by epithelial apoptosis and moderate TJ changes in HT-29/B6 cells [9]. The study also supported the pathogenetic principle of a paracellular leak-flux mechanism exhibited by *C. concisus* to induce diarrhea. However, a clinical epidemiological observation found that *C. concisus*-infected patients present prolonged watery diarrhea with the milder intestinal inflammatory outcome and less fever compared to *C. jejuni*-infected patients [8]. The symptom of watery diarrhea correlates with our experimental finding that *C. concisus* impairs ENaC-dependent sodium absorption in the distal colon leading to watery rather than bloody diarrhea which is frequently induced by other cytotoxic enteropathogens. This feature of *C. concisus* infection was also reflected by our experimental findings of unchanged cell viability with retention of active Cl^−^ secretion, defined TJ changes with claudin-8 dysregulation, and no induction of lesions or cytotoxic destruction of the tissue after *C. concisus* infection.

As the second main result, we showed that *C. concisus* induced ERK activation in HT-29/B6-GR/MR cell monolayers. In the corresponding blockade experiment, ERK inhibition with U0126 ameliorated ENaC dysfunction after *C. concisus* infection, which is direct evidence that *C. concisus* infection impaired ENaC function via ERK activation. This means the bacteria not only caused general cell damage, but they also decreased ENaC function via ERK activation. Moreover, a reduction in the mRNA expression of regulatory ENaC subunits (-β and -γ) 48 h after *C. concisus* infection (Figure 3) indicated that *C. concisus* dysregulates ENaC function. Similar mechanisms of functional ENaC dysregulation were previously reported in Crohn’s disease and ulcerative colitis as well as for lymphocytic colitis [18,19,20].

**Figure 11:**
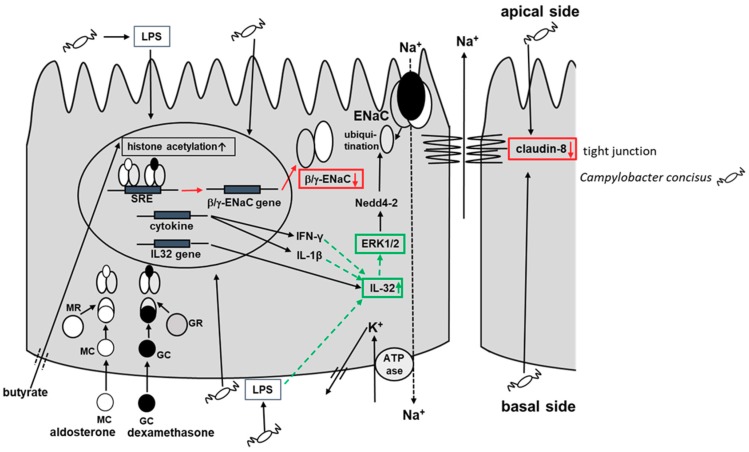
Scheme of *Campylobacter concisus*-induced impairment of ENaC-dependent Na^+^ absorption in colonic epithelial cells. In diarrheal state, glucocorticoid and mineralocorticoid receptors (GR and MR) are activated by glucocorticoids (GC) like dexamethasone and mineralocorticoids (MC) like aldosterone respectively. GR and MR forms a heterodimer, binds to steroid-responsive element (SRE) and activates the expression of β/γ ENaC genes with the subsequent protein synthesis to enhance the electrogenic Na^+^ absorption. Butyrate inhibits histone deacetylation to intensify the expression of β/γ ENaC genes. GC (dexamethasone), MC (aldosterone) and butyrate were the activators of ENaC used in our experimental setup [17]. From the findings of our study, the red arrows in the figure represent *C. concisus*-induced transcriptional down-regulation or dysregulation of protein synthesis. The green arrows in the figure represent the transcriptional up-regulation and activation of signaling pathways (ERK pathway) by *C. concisus*. *C. concisus* promoted the transcriptional up-regulation of interleukin-32 (IL-32), which might increase its protein expression (indicated by upward green arrow) and lead to activation of neural precursor cell expressed developmentally down-regulated protein (NEED4-2)-dependent ubiquitination of ENaC (via ERK) and impair ENaC-dependent Na^+^ absorption. The dotted green lines represent the predicted activation of IL-32 by upstream regulators IFN-γ, IL-1β and bacterial LPS (lipopolysaccharides), which could contribute to ERK activation leading to ENaC dysfunction in DBA-stimulated controls 48 h after *C. concisus* infection (bioinformatics prediction from RNA-Seq data by Ingenuity Pathway Analysis (IPA) software). The mRNA expression of NEED4-2 in the HT-29/B6-GR/MR cell model is confirmed through RNA-Seq data. However, the regulation of NEDD4-2 via ERK1/2 and ubiquitination of ENaC which might lead to disassembly of ENaC subunits from epithelium is also a prediction through IPA. The reduction in claudin-8 protein expression by *C. concisus* is indicated by red color. The reduction in claudin-8 expression and protein redistribution perturbs the ionic paracellular barrier and leads to back leakage of Na^+^ into the apical side, contributing to the net loss of Na^+^.

Tumor necrosis factor (TNF)-α was identified as an important pro-inflammatory cytokine that could down-regulate colonic ENaC expression in different studies [18,30]. *C. concisus* also induced the release of pro-inflammatory cytokines such as interleukin-8 and TNF-α from intestinal epithelial cells, macrophages and/or THP1 immune cells [31]. Hence, we presumed that TNFα-mediated ERK activation might contribute to ENaC dysfunction in *C. concisus* infection, as previously demonstrated for Crohn’s disease [19] and lymphocytic colitis [20]. However, our RNA-Seq analysis indicated that IL-32 is the cytokine with the highest mRNA expression change rather than TNF-α in *C. concisus* infection. The bioinformatics prediction through Ingenuity Pathway Analysis (IPA) from our RNA-Seq data indicated that *C. concisus* could promote ERK activation via IL-32, which might lead to ENaC dysfunction in TNF-α-independent pathway (scheme, Figure 11). IL-32 has been reported to induce activation of ERK in fibroblast-like synoviocytes in rheumatoid arthritis [32] and human calcified aortic valves [33].

Interestingly, IL-32 can also be activated by interferon-γ (IFN-γ) and interleukin-1β (IL-1β) or through bacterial lipopolysaccharides (LPS) according to our Ingenuity Pathway Analysis (IPA) analysis (Supplementary Table S3). Thus, it may be reasonable to conclude that in the presence of the sub-epithelial immune compartment, cytokines like IFN-γ and IL-1β released in response to *C. concisus* infection could intensify the ENaC dysfunction observed in the HT-29/B6-GR/MR cell model in our present study.

We used amiloride, which selectively inhibited apical ENaC-mediated Na^+^ entry. In parallel with the initial aldosterone-dependent and ENaC-mediated increase and the subsequent amiloride-dependent inhibition of Na^+^ absorption, an initial decrease and subsequent amiloride-induced increase in TER were observed. These changes in TER simply reflect the opening and closure of the ENaC and do not give any information on the effect of *C. concisus* infection on the paracellular barrier function. However, the overall TER after amiloride directly reflects the integrity of the paracellular barrier in HT-29/B6-GR/MR cell monolayer. Thus, a direct comparison becomes possible between infected and control monolayers. In this context, TJ protein claudin-8 plays a crucial role as it seals the paracellular space, in order to prevent the back leakage of Na^+^ into the apical compartment [25].

The third important finding of our study shows that *C. concisus*-induced Na^+^ malabsorption is accompanied by a decrease in TER, mediated by a reduction in the mRNA and protein expression of claudin-8. This might point to a paracellular barrier dysfunction promoted by *C. concisus* either as a parallel or as a subsequent effect to ENaC dysfunction. Moreover, a redistribution of claudin-8 from the TJ domain of the cells to intracellular compartments was observed after *C. concisus* infection. Hence, we could confirm that *C. concisus* impairs Na^+^ absorption in colonocytes not only by ENaC dysfunction but also by claudin-8 disruption, leading to a loss of Na^+^ via the paracellular pathway (scheme, Figure 11). A possible explanation for the proposed paracellular barrier dysfunction promoted by *C. concisus*, comes from one of our previous studies on claudin-8 regulation in response to ENaC activity [25], which revealed that ENaC stimulation also induces claudin-8 expression. Hence, we could ascertain that functional ENaC impairment by *C. concisus* could contribute to claudin-8 expression changes and might contribute to paracellular barrier dysfunction.

Previously, claudin-8 down-regulation was determined in *C. jejuni* infection in the human colon mucosa. However, this had not been linked with Na^+^ malabsorption and was rather discussed in the context of a general pro-inflammatory barrier dysfunction [26]. From the findings of our present study on *C. concisus*, a specific contribution of claudin-8 to the loss of Na^+^ seems much more likely. Very few claudins exist with paracellular channel function, like claudin-2 and -15 which are predominantly expressed in proximal intestinal segments like the jejunum. Other claudins like claudin-8 have rather sealing functions. Together with the expression of distinct transport proteins in the plasma membrane of the enterocytes, TJ proteins define the properties of a specific intestinal segment to be leaky or tight. Thus, a co-regulation of specific claudins and the corresponding channels is predictable but not yet shown. The signaling connection between MAPK and claudin-8 was shown in *Yersinia enterocolitica* infection and JNK [34] and colorectal cancer and ERK [35]. From this, we can hypothesize that the ENaC and claudin-8 co-regulation could happen via ERK/MAPK in *Campylobacter* infection. However, this should be confirmed only after a detailed investigation.

In our present study, a similar pathomechanism was seen after *C. concisus* infection as previously described in lymphocytic colitis, in which claudin-8 disruption and ENaC dysfunction synergistically promote watery diarrhea [19,36]. Interestingly, a clinical study found that 12% of *C. concisus*-infected patients with prolonged diarrhea developed microscopic colitis (lymphocytic colitis is a subtype of microscopic colitis) in a six-month follow-up period [37]. We also observed a significant increase in the expression of the TJ protein occludin in HT-29/B6-GR/MR cell monolayers after infection with *C. concisus*. This could be a result of host cell autophagy modulation required for intracellular survival of *C. concisus* [38,39]. Besides, occludin was demonstrated not to affect the ionic barrier properties of intestinal epithelia in an occludin knockout mouse model, since the transepithelial electrical resistance did not differ between occludin-deficient mice and their wild-type littermates [40,41]. Hence, the increase in occludin expression has to be interpreted as an independent phenomenon regardless of the paracellular barrier change induced by *C. concisus*.

In order to confirm the effects of *C. concisus* on ENaC function in vivo, we employed the abiotic IL-10^−/−^ mouse model. Mice display a strong physiological colonization resistance of the intestine due to the mouse-specific gut microbiota composition and are therefore protected from infection with enteropathogens including *C. jejuni* [42,43]. Furthermore, mice are *per se* approximately 10,000-fold more resistant to LOS and lipopolysaccharide (LPS), the major cell wall constituents of *C. jejuni* or other Gram-negative bacteria [44,45] as compared to humans [46]. It was recently shown that the abiotic IL-10^−/−^ mice (gut microbiota depleted by broad-spectrum antibiotic treatment) are effectively colonized by *C. jejuni* upon peroral infection and develop key features of acute human Campylobacteriosis [27]. The main reasons for these severe *C. jejuni-*induced immunopathological responses in the acute stages of enterocolitis in mice are (i) the absence of colonization resistance following microbiota depletion and (ii) the lack of IL-10 enhancing susceptibility of mice to *C. jejuni* LOS [27,47]. In consequence, abiotic IL-10^−/−^ mice infected with *C. jejuni* display a pronounced LOS-induced and Toll-like receptor (TLR)-4-dependent innate and adaptive immune response in the intestine [27]. Since then, abiotic IL-10^−/−^ mouse model has been successfully employed in many studies [48,49,50,51]. In recent studies, the abiotic IL-10^−/−^ mouse model was also used to determine the barrier protective and anti-inflammatory effects on *C. jejuni* infection using curcumin or vitamin D [52,53].

The first experimental infection of mice by *C. concisus* to study inflammatory effects on the intestine was carried out in Bagg Albino/c (BALB/c) mice [54]. However, this study reported poor colonization of *C. concisus* in the intestine of BALB/c mice without any substantial inflammation and proposed an improved mouse model from a different mouse strain for future investigations. In the current study, we achieved successful bacterial colonization of the colon in *C. concisus*-infected abiotic IL-10^−/−^ mice (C57BL/6 strain). Further, in concordance to our in vitro model, a decrease in ENaC-dependent Na^+^ transport could be measured in the colon of *C. concisus*-infected IL-10^−/−^ mice 6 days post-infection. This gives us a solid piece of evidence for *C. concisus*-induced ENaC dysfunction in vivo. However, we have not observed any differences in R^epi^ and R^sub^ values between the colon mucosa of controls and *C. concisus*-infected mice. This indicates that *C. concisus* neither induces massive inflammation nor impairs epithelial barrier function in the colon but can still cause ENaC dysfunction.

Taken together, *C. concisus* impairs ENaC-dependent Na^+^ absorption via down-regulation of β- and γ-ENaC mRNA expression and ERK activation. The mRNA expression of pro-inflammatory cytokine IL-32 is up-regulated after *C. concisus* infection, which might contribute to ERK activation, in turn leading to ENaC dysfunction. In parallel, *C. concisus* disrupts claudin-8 and facilitates back leakage of Na^+^ ions. Thus, *C. concisus* induces ENaC dysfunction via ERK activation and claudin-8-dependent barrier dysfunction—both of which contribute to Na^+^ malabsorption and diarrhea.

## 4. Materials and Methods

### 4.1. Cell Culture and Campylobacter Infection

HT-29/B6-GR/MR cell monolayers (epithelial cell line HT-29/B6 transfected with glucocorticoid and mineralocorticoid receptors; [15]) were used to determine functional ENaC activity in vitro. Fresh HT-29/B6-GR/MR cells were cultured in Roswell Park Memorial Institute (RPMI) medium (Sigma Aldrich, St. Louis, MO, USA) for one week at 37 °C in humidified atmosphere (95% air/5% CO_2_). RPMI media were supplemented with 10% fetal calf serum (FCS; Gibco, Carlsbad, CA, USA), 1% penicillin/streptomycin (Gibco, Carlsbad, CA, USA), 500 IU/mL G418 (Merck Millipore, Billerica, MA, USA) and 200 μg/mL hygromycin B (Biochrom GmbH, Berlin, Germany). After one week, trypsinized cells were seeded on Millicell PCF filters of 3 μm pore size (Merck Millipore, Billerica, MA, USA) and cultured for 7–10 days. Experiments were performed when cell monolayers reached a transepithelial electrical resistance (TER) of 1500–2100 Ω·cm^2^. Cells were incubated with 10% hormone-free FCS (h-f FCS; Sigma-Aldrich, St. Louis, MO, USA) for 24 h and stimulated with DBA (a combination of dexamethasone (D, 50 nM; Sigma-Aldrich, St. Louis, MO, USA), Na^+^ butyrate (B, 2 mM; Merck-Schuchardt, Hohenbrunn, Germany) and aldosterone (A, 3 nM; Sigma-Aldrich, St. Louis, MO, USA)) for four days.

**Figure 12:**
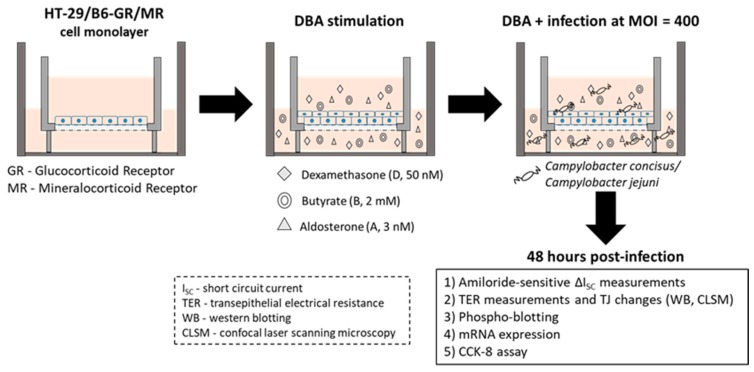
Study design to determine ENaC activity and tight junctional changes after *Campylobacter concisus* or *Campylobacter jejuni* infections using the HT-29/B6-GR/MR in vitro cell model.

Three days after DBA stimulation, cell monolayers were washed and incubated with heat-inactivated 10% h-f FCS without any antibiotic supplements for 24 h. Four days post-DBA stimulation, TER values were recorded with chop-stick electrodes and cell monolayers were infected with *C. concisus* (*C. concisus* AAuH 37 UC oral [55]) or *C. jejuni* (*C. jejuni* wild-type strain 81–176) on both apical and basolateral sides of the cell monolayers at a multiplicity of infection (MOI) of 400 (Figure 12). After infection, cell monolayers were incubated in a H_2_-containing atmospheric condition [microaerophilic/CO_2_-enriched gas pack (BD GasPak EZ CampyPak container system sachets, BD Biosciences, San Jose, CA, USA) and 10% hydrogen gas 0.082 g of sodium borohydride (NaBH_4_) in 10 mL of distilled water in 2.5 L airtight plastic jar] at 37 °C for approximately 30 h. Cell monolayers were then placed in a humidified atmosphere at 37 °C. Forty-eight hours post-infection, cell monolayers were used for amiloride-sensitive short circuit current (I_SC_) measurements in Ussing chambers, total RNA isolation, Western blot analysis, confocal laser-scanning microscopy (CLSM, Zeiss LSM 780, Jena, Germany) and CCK-8 assay.

### 4.2. Electrophysiological Determination of ENaC Function In Vitro

Forty-eight hours post-infection with *Campylobacter* spp., HT-29/B6-GR/MR cell monolayers grown on filters were mounted in Ussing chambers (epithelial surface area of 0.6 cm^2^; Institute of Clinical Physiology, Charité, Berlin). The composition of the bathing solution in the Ussing chambers was as follows: Na^+^ 140.0 mM; Cl^−^ 123.8 mM; K^+^ 5.4 mM; Ca^2+^ 1.2 mM; Mg^2+^ 1.2 mM; HPO_4_^2−^ 2.4 mM; H_2_PO_4_^−^ 0.6 mM and HCO^3−^ 21.0 mM. The solution was gassed with carbogen gas (95% O_2_ and 5% CO_2_) by bubble lift. Temperature was maintained at 37 °C, pH 7.4. TER (Ω·cm^2^) and short circuit current (I_SC_; µA/cm^2^) were recorded using voltage clamp devices (CVC6, Fiebig Hard & Software, Berlin, Germany). Cell monolayers were allowed to stabilize and ENaC-dependent Na^+^ transport recorded as a decrease in I_SC_ (∆I_SC_; µA/cm^2^) 20 min after the apical addition of the ENaC blocker amiloride (100 µM; Sigma-Aldrich, St. Louis, MO, USA). For the complete inhibition of ENaC in the colonic epithelium and mucous-producing HT-29/B6-GR/MR cells, which is covered by a mucous layer, an amiloride concentration of 100 µM was employed. This concentration is ten-fold higher than concentrations usually used to completely block the ENaC in kidney cell models (10 µM), but still specific for Na^+^ transport via the ENaC, as NHE3, the other transport system for Na^+^ in the apical cell membrane of colonocytes, is only affected by amiloride concentrations at 1 mM [17]. To ensure that epithelial cells were functionally viable during ∆I_SC_ measurements, electrogenic chloride Cl^−^ secretion by the cells was determined by measuring the increase in I_SC_ at end of each experiment in response to the addition of theophylline (10 mM) and PGE2 (10 µM), or carbachol (100 µM).

### 4.3. Western Blot Assessment of Tight Junction Protein Expression

Forty-eight hours post-infection, TERs of cell monolayers were recorded 20 min after measuring the amiloride-induced changes in I_SC_. Cell monolayers of HT-29/B6-GR/MR were then prepared prior to evaluating changes in TJ protein expression. Control and infected cells were scraped carefully from the cell monolayers and subjected to total cell lysis using a lysis buffer (150 mM NaCl, 10 mM Tris buffer pH of 7.5, 0.5% Triton X-100, and 1% SDS). The concentration of the proteins isolated was estimated by the Pierce bicinchoninic acid (BCA) assay (Thermo Scientific, Waltham, MA, USA) according to manufacturer’s instruction. Proteins were resolved using 12.5% SDS-PAGE gel, and 15 µg of proteins were used from each sample. The resolved proteins were electro-transferred to PVDF nitrocellulose membranes (Thermo Scientific, Waltham, MA, USA) using the Trans-Blot system (Bio-Rad Laboratories, Inc., Hercules, CA, USA) at 25 V for 15–17 min.

PVDF membranes were subjected to incubation, shaking with a blocking solution containing 1% polyvinylpyrrolidone (PVP-40; Sigma Aldrich, St. Louis, MO, USA) in tris-buffered saline (TBS) supplemented with 0.05% Tween-20 buffer at room temperature (RT) for 2 h to avoid unspecific protein signals. Membranes were incubated with primary antibodies rabbit (Rb) anti-claudin-1, -2, -5, -8 (Invitrogen, Carlsbad, CA, USA), Rb anti-occludin (Sigma Aldrich, St. Louis, MO, USA), Rb anti-tricellulin (Invitrogen, Carlsbad, CA, USA), mouse (M) anti-β-actin (Sigma Aldrich, St. Louis, MO, USA), M anti-GAPDH (Merck KGaA, Darmstadt, Germany) overnight at 4 °C. Membranes were then incubated with appropriate secondary antibodies (peroxidase-conjugated goat anti-Rb and goat anti-M, Jackson ImmunoResearch, Ely, UK) at RT for 2 h. Membranes were evaluated for bands of specific protein with a chemiluminescence solution (Thermo Scientific, Waltham, MA, USA) using the FUSION FX7 system (Vilber Lourmat Deutschland GmbH, Eberhardzell, Germany). Protein bands were quantified by ImageJ software (Rasband, W. S., ImageJ, National Institute of Health (NIH), Bethesda, MD, USA). Densitometric analysis of the Western blots was performed by normalizing the band intensity of TJ proteins to their respective β-actin or GAPDH band intensities.

### 4.4. Western Blot Assessment of ERK Phosphorylation

Forty-eight hours post-infection, HT-29/B6-GR/MR cell monolayers were washed and incubated with RPMI media lacking h-f FCS and supplemented with 2 mg/mL gentamycin (Gibco, Carlsbad, CA, USA) for 3 h to remove and kill all residual bacteria on the apical and basal sides of the cell monolayers. Cell monolayers were then washed with heat-inactivated 10% h-f FCS to completely remove gentamycin, after which cells were DBA stimulated and re-infected with *C. concisus* or *C. jejuni* for 5, 15, 30, 60 and 120 min before detecting protein phosphorylation by Western blotting. Cells were scraped carefully from control and the infected monolayers, and removed using complete cell lysis buffer (pH 7.5) supplemented with phosphatase inhibitors (20 mM Tris, 150 mM NaCl, 1 mM Triton X-100, 1 mM EDTA, 1 mM PMSF, 2.5 mM Na^+^ pyrophosphate, 1 mM β-glycerophosphate, 1 mM Na^+^ orthovanadate, 1 mM EGTA, 1µg/mL leupeptin, complete protease inhibitor cocktail (Roche, Mannheim, Germany)).

Proteins were isolated from lysed cells and their concentrations determined. Proteins were then resolved and electro-transferred to PVDF nitrocellulose membranes (Thermo Scientific, Waltham, MA, USA). PVDF membranes were blocked, incubated with primary and secondary antibodies and evaluated for specific proteins using FUSION FX7, as described in the Section 4.3. Primary antibodies used to determine ERK phosphorylation were Rb anti-p-ERK1/2, Rb anti-ERK1/2 (Cell Signaling Technology Europe B.V., Frankfurt am Main, Germany) and M anti-β-actin (Sigma Aldrich, St. Louis, MO, USA). Densitometric analysis of Western blots was performed by normalizing band intensities of p-ERK1/2 and ERK1/2 (total ERK1/2) to their respective β-actin intensities.

### 4.5. Functional Blockade of ERK to Determine the Changes in ENaC Function In Vitro

Upstream MEK inhibitor U0126 (Biogems International, Inc. Westlake Village, CA, USA), which functionally blocks ERK activity, was used to determine the changes in ENaC-dependent Na^+^ transport (∆I_SC_) after *C. concisus* infection in HT-29/B6-GR/MR cells. For this purpose, we used the in vitro infection model as described in Figure 11 and Section 4.1 with few modifications. Four days post-DBA stimulation, the apical and basolateral compartments of the cell monolayers were treated with the functional MEK inhibitor U0126 at a concentration of 10 µM supplemented along with heat-inactivated 10% h-f FCS without any antibiotic supplements. Then, the cell monolayers were incubated at 37 °C in humidified atmosphere (95% air/5% CO_2_) for 2 h. Then, the cell monolayers were infected with *C. concisus* on both apical and basolateral compartment of the cell monolayers at MOI of 400. Following infection, the cell monolayers were incubated in a special microaerobic atmospheric condition (as described in Section 4.1) but only for approximately 4 h. Then, the cell monolayers were replaced at 37 °C in humidified atmosphere. Forty-eight hours post-infection, ENaC-dependent Na^+^ transport (∆I_SC_) was determined as described in Section 4.1.

### 4.6. ENaC Regulatory β- and γ-Subunit and Claudin-8 mRNA Expression Analyzed by RT-qPCR

Total RNA was extracted from HT-29/B6-GR/MR cells using the mirVana^TM^ miRNA Isolation Kit (Ambion, Life Technologies, Carlsbad, CA, USA). cDNA was synthesized by reverse-transcription PCR using the High-Capacity cDNA Archive Kit (Applied Biosystems, Mannheim, Germany) with oligo(dT) primer. Real-time PCR was performed according to the manufacturer’s instructions with an 7500 FAST Real-Time PCR System (Applied Biosystems, Mannheim, Germany) device using the TaqMan^®^Gene Expression protocol [HS00165722_m1 for human ENaC β-subunit (*SCCN1B*), HS00168918_m1 for human ENaC γ-subunit (*SCNN1G*), HS00273282_s1 for human claudin-8 (*CLDN8*)] with FAM™dye-labeled primers. GAPDH-cDNA was quantified using VIC^®^reporter dyes as endogenous control (all Applied Biosystems, Mannheim, Germany). Differential gene expression was determined by the 2^−ΔΔCT^ method [56] and represented as fold-induction with respect to controls.

### 4.7. Tight Junction Protein Localization Evaluated by Immunofluorescence and Confocal Laser Scanning Microscopy

TJ protein distribution in HT-29/B6-GR/MR cell monolayers was investigated four days post-DBA stimulation and 48 h post-infection with *Campylobacter* spp. Cell monolayers on 3 μM PCF filters were fixed using 2% paraformaldehyde (PFA; Electron Microscopy Sciences, Hatfield, PA, USA) at RT for 20 min. After fixing, cell monolayers were quenched with 25 mM Glycin (Biomol GmBH, Hamburg, Germany), washed twice with phosphate-buffered saline (PBS; with Ca^2+^/Mg^2+^; pH 7.4; Sigma Aldrich, St. Louis, MO, USA) and permeabilized with 0.5% Triton X-100 (Sigma Aldrich, St. Louis Missouri, MO, USA) for 7 min at RT. Permeabilized monolayers were then washed and incubated with a blocking solution (1% (*v*/*v*) goat serum, Gibco, Carlsbad, CA, USA; diluted with Ca^2+^- and Mg^2+^-containing PBS) at RT for 30 min. After blocking, cell monolayers were incubated for 45 min at 37 °C with the primary antibodies Rb anti-claudin-8 (Thermo Scientific, Waltham, MA, USA) and M anti-human ZO-1 (BD Biosciences, Franklin Lakes, NJ, USA) at their optimal concentrations.

Following this, cell monolayers were washed twice with 1% goat serum and incubated with secondary antibodies diluted at concentration of 1:400 in blocking solution (goat anti-Rb green, Alexa Fluor Plus 488 nm and goat anti-M red, Alexa Fluor 594 nm (Invitrogen Carlsbad, CA, USA)) for 45 min at 37 °C. After incubation with secondary antibodies, monolayers were stained for nuclei with 4′-6-diamidino-2-phenylindole dihydrochloride (DAPI; Roche AG, Basel, Switzerland) at a dilution of 1:1000 in blocking solution. Monolayers were then washed with blocking solution and Ca^2+^- and Mg^2+^-containing PBS and rinsed briefly with water and absolute ethanol. The cell filters were dried and mounted on glass slides using the mounting solution ProTaq Mount Fluor (Biocyc, Luckenwalde, Germany), and fixed with coverslips. Localization and/or redistribution of TJ protein claudin-8 (co-stained with ZO-1) in control and the infected cell monolayers was determined by confocal laser-scanning microscopy (CLSM, Zeiss LSM 780, Jena, Germany). Individual Z-stacks of the cell monolayers were recorded using the laser scan function.

### 4.8. Electrophysiological Determination of ENaC Function in an In Vivo Model of C. concisus Infection

IL-10^−/−^ mice were used as in vivo model of *C. concisus* infection. IL-10^−/−^ mice (in C57BL/6j background) were held under specific pathogen-free (SPF) conditions in the animal facilities of the Forschungseinrichtung für Experimentelle Medizin (Charité—Universitätsmedizin Berlin). Mice were transferred to sterile cages and treated for eight weeks with an antibiotic cocktail in the drinking water ad libitum [supplemented with ampicillin/sulbactum (1.5 g/L), ciprofloxacin (200 mg/L), impenim/cilastatin (250 mg/L)] to remove the commensal gut bacteria. Mice were then infected with *C. concisus* (*C. concisus* AAuH 37 UC oral; [55]) via oral gavage at 10^8^ colony-forming units (CFU) in a volume of 0.3 mL PBS. Mice infected with commensal *E. coli* at same CFU were used as controls. Six days after infection, animals were sacrificed by isoflurane inhalation and their colons carefully removed.

The distal parts of the colon were mounted in Ussing chambers and equilibrated with the bathing solution (Na^+^ 140.0 mM; Cl^−^ 123.8 mM; K^+^ 5.4 mM; Ca^2+^ 1.2 mM; Mg^2+^ 1.2 mM; HPO_4_^2−^ 2.4 mM; H_2_PO_4_^−^ 0.6 mM and HCO^3−^ 21.0 mM) for approximately 30 min. The solution was gassed with carbogen gas (95% O_2_ and 5% CO_2_) by bubble lift. Temperature was maintained at 37 °C, pH 7.4. Transepithelial resistance (TER, Ω·cm^2^) and short circuit current (I_SC_, µA/cm^2^) were recorded using voltage clamp devices (CVC6, Feibig Hard & Software, Berlin, Germany). Distal colons were then treated with the mineralocorticoid aldosterone (3 nM) added to both the apical and basolateral compartments of the Ussing chambers to stimulate ENaC activity. Six hours later, ENaC-dependent Na^+^ transport was determined as the decrease in I_SC_ 15 min after the addition of the ENaC blocker amiloride (100 µM; Sigma-Aldrich, St. Louis, MO, USA) to the apical compartment. To confirm viability of the colonic epithelium after 6 h of aldosterone exposure, the Cl^−^ secretory response of the epithelium was assessed by measuring increases in I_SC_ at end of each experiment after the addition of theophylline (10 mM) and prostaglandin E_2_ (10 µM). Subsequently, inhibition of the stimulated Cl^−^ secretion was assessed by measuring decreases in I_SC_ after the addition of bumetanide (100 µM) to the basolateral compartment.

In both control and inflamed colon, I_SC_ measurements could be influenced to different degrees by the thickness of the subepithelial tissue layers. Thus, in addition to I_SC_ measurements, the total transepithelial resistance (R^total^) and subepithelial resistance (R^sub^) of colonic samples were recorded via one-path impedance spectroscopy, as described previously [57]. Epithelial resistance (R^epi^) was determined by subtracting R^sub^ from R^total^. To ensure that changes in Isc accurately reflected changes in active transport, the contributions from subepithelial tissue were taken into account by calculating the ratio R^total^/R^epi^, as described previously [20,26].

### 4.9. Ethics Statement

Animal experiments were carried out in the animal facility at the Forschungseinrichtung für Experimentelle Medizin (Charité—Universitätsmedizin Berlin) according to the German animal protection law (approval number G0172/16 (13 October 2016), LaGeSo Berlin).

### 4.10. RNA-Seq Expression Analysis

Total RNA was obtained from HT-29/B6-GR/MR cells using the mirVana^TM^ miRNA Isolation Kit (Ambion, Life Technologies, Carlsbad, CA, USA). RNA sequencing was performed using the TrueSeq Stranded Total RNA method on a NovaSeq^TM^ 6000 Sequencing System (https://www.illumina.com/) with quality scores of ≥80%.

The reads from RNA-Seq were mapped against the human genome GRCh38 release 97 and sorted using the STAR aligner version 2.7.1a in a two-pass mode [58]. First-pass read mapping utilized coordinates from Ensembl annotation release 97 as a framework. Second-pass mapping added splice sites that were found in the first run. Count tables containing gene-read coverages were obtained using the feature Counts function of the Bioconductor package Rsubread [59], with coordinates from aforementioned Ensembl annotation and default parameters.

The Bioconductor package DESeq2 [60] was used to quantify the differential expression of genes between two conditions in form of log2-fold changes with their corresponding *p*-values. *p*-values were adjusted for multiple testing using the Benjamini–Hochberg procedure. Pathway analysis was performed with Ingenuity Pathway Analysis software (IPA, Qiagen Silicon Valley, Redwood, CA, USA) to evaluate the *C. concisus*-dependent changes in the expression of different genes that regulate ENaC function. Fastq files containing the unprocessed raw reads from sequencing and a raw counts matrix table are publicly available at Gene Expression Omnibus (GEO) archive under National Centre for Biotechnology Information (NCBI) website with GEO accession ID 141217 [*Campylobacter concisus* impairs sodium absorption via ENaC dysfunction and claudin-8 disruption. Available online: https://www.ncbi.nlm.nih.gov/gds/?term=GSE141217 (1 January 2020)].

In addition, counts per million and log-transformed counts per million (CPM) normalization was performed using CPM function of the Bioconductor package edgeR [61], and the gene expression of IL-32 was determined using CPM.

### 4.11. Cell Proliferation and Cytotoxicity Assay

The possibility of cytotoxicity and the cell proliferation rate in HT-29/B6-GR/MR cell monolayers 48 h after *C. concisus* infection were evaluated by CCK-8 assay (Cell Counting Kit-8, Thermo Scientific, Waltham, MA, USA). HT-29/B6-GR/MR cells were seeded into 96 well plates and incubated for 7–10 days at 37 °C in a humidified atmosphere (95% air/5% CO_2_). Cells were stimulated with DBA (a combination of dexamethasone (D, 50 nM; Sigma-Aldrich, St. Louis, MO, USA), Na^+^ butyrate (B, 2 mM; Merck-Schuchardt, Hohenbrunn, Germany) and aldosterone (A, 3 nM; Sigma-Aldrich, St. Louis, MO, USA)) for four days after an overnight incubation with 10% hormone-free FCS (h-f FCS; Sigma-Aldrich, St. Louis, MO, USA). Four days post-DBA stimulation, cells in 96 well plates were infected with *C. concisus* (*C. concisus* AAuH 37 UC oral; [43]) at a multiplicity of infection (MOI) of 400. Forty-eight hours post-infection, cell viability was determined by CCK-8 assay according to manufacturer’s instructions. 10 µL of CCK-8 solution (WST-8 [2-(2-methoxy-4-nitrophenyl)-5-(2, 4-disulfophenyl)-2H-tetrazolium, monosodium salt]) was added to 100 µL of cell suspensions in 96 well plates. WST-8 produced a water-soluble formazan dye (orange colored product) generated by oxidation of cellular dehydrogenases [62,63]. Two hours after addition of the CCK-8 solution to the 96 well plates, absorbance values were recorded using a spectrophotometer (Tecan GmbH, Maennedorf, Switzerland) at 450 nm, with a reference wavelength of 600 nm. Absorbance values represented the amount of formazan dye generated by cellular dehydrogenases and were directly proportional to the number of living cells.

### 4.12. Statistical Analysis

All data are expressed as the mean value ± standard error of the mean (SEM). Statistical analyses were performed with GraphPad Prism (GraphPad Software version 5.0, Inc., San Diego, CA, USA). For data in Figure 1, Figure 3, Figure 4, Figure 5, Figure 6, Figure 7 and Figure 9, the unpaired t-test with Welch’s correction for unequal variances was applied. For data that were not normally distributed (Figure 10 and Table 1), the Mann–Whitney *U*-Test was used. To compare data sets from three different samples (data of Figure 2), two-way ANOVA with Bonferroni–Holm adjustment was used. *p* < 0.05 was considered statistically significant.

## 5. Conclusions

*Campylobacter concisus* impairs ENaC-dependent Na^+^ absorption via down-regulation of β- and γ-ENaC mRNA expression and ERK activation. The up-regulated mRNA expression of pro-inflammatory cytokine IL-32 after *C. concisus* infection might contribute to ERK activation, in turn leading to ENaC dysfunction. Besides, *C. concisus* disrupts claudin-8 and facilitates back leakage of Na^+^ ions. Hence, *C. concisus* induces ENaC dysfunction via ERK activation and claudin-8-dependent barrier dysfunction—both of which contribute to Na^+^ malabsorption and diarrhea.

## Figures and Tables

**Table 1:** Evaluation of colonic viability.

Abiotic IL-10^−/−^ Mice	∆I_SC_ (µA/cm^2^) after PGE2 + Theophylline	∆I_SC_ (µA/cm^2^) after Bumetanide	Resistance		
			R^epi^	R^sub^	R^total^
Control (*n* = 4–8)	27 ± 7.99	−15 ± 4.08	28.4 ± 3.62	24.8 ± 2.15	52.4 ± 3.55
*C. concisus*-infection (*n* = 5–8)	25 ± 8.12	−16 ± 5.57	33.2 ± 3.10	28.5 ± 2.57	61.7 ± 3.90
Significance	ns	ns	ns	ns	ns

ns, not significant; PGE2, prostaglandin E_2_; R^total^, total transepithelial resistance; R^sub^, subepithelial resistance; R^epi^, epithelial resistance. Data represent the mean ± SEM (*p* > 0.05, ns). No significant difference in correction factors (R^total^/R^epi^) for active transport rates (i.e., I_SC_) were observed between controls and *C. concisus*-infected mice [controls = 1.99 ± 0.18 and *C. concisus* = 1.91 ± 0.10, *n* = 8, *p* > 0.05, ns].

## Supplementary Materials

The following are available online at https://www.mdpi.com/1422-0067/21/2/373/s1, Figure S1: mRNA expression changes of ENaC subunits (-α, -β, -γ) in HT-29/B6-GR/MR cells 48 h after *Camplyobacter concisus* infection; Table S1: Differentially expressed genes in C. concisus infection; Table S2: mRNA expression of different ion-channel transporters after *C. concisus* infection; Table S3: Upstream regulator analysis.

## Abbreviations

cAMP	Cyclic adenosine monophosphate
cDNA	Complementary DNA
CFU	Colony forming unit
CLSM	Confocal laser scanning microscopy
DAPI	4′-6-diamidino-2-phenylindole dihydrochloride
DBA	Dexamethasone, butyrate and aldosterone
EDTA	Ethylenediaminetetraacetic acid
EGTA	Ethylene glycol-bis(β-aminoethyl ether)-*N*,*N*,*N*′,*N*′-tetraacetic acid
ENaC	Epithelial sodium channel
ERK	Extracellular signal-regulated kinase
FCS	Fetal calf serum
GAPDH	Glyceraldehyde 3-phosphate dehydrogenase
GC	Glucocorticoids
GR	Glucocorticoid receptor
H_2_PO_4_^−^	Dihydrogen phosphate
HCO_3_^−^	Hydrogen carbonate
h-f FCS	Hormone-free FCS
HPO_4_^2−^	Hydrogen phosphate
IFN	Interferon
IL	Interleukin
I_SC_	Short circuit current
JAM	Junctiunal adhesion molecule
JNK	c-Jun N-terminal kinase
LaGeSo	Landesamt für Gesundheit und Soziales
MAPK	Mitogen-activated protein kinase
MC	Mineralocorticoids
MEK	Mitogen-activated protein kinase kinase
miRNA	MicroRNA
MOI	Multiplicity of infection
MR	Mineralocorticoid receptor
mRNA	Messenger RNA
PBS	Phosphate-buffered saline
PGE2	Prostaglandin E2
PMSF	Phenylmethylsulfonyl fluoride
PVDF	Polyvinylidene fluoride
PVP	Polyvinylpyrrolidone
STAT-6	Signal transducer and activator of transcription 6
TER	Transepithelial electrical resistance
TJ	Tight junction
TNF	Tumor necrosis factor
