# Supplementary material for: Campylobacter concisus Impairs Sodium Absorption in Colonic Epithelium via ENaC Dysfunction and Claudin-8 Disruption
Source: Int J Mol Sci. 2020 Jan 7;21(2):373. doi: 10.3390/ijms21020373 (PMC7013563; doi:10.3390/ijms21020373)
Supplement: Supplementary file 1 [file ijms-21-00373-s001.zip › Supplementary Figure S1.docx]

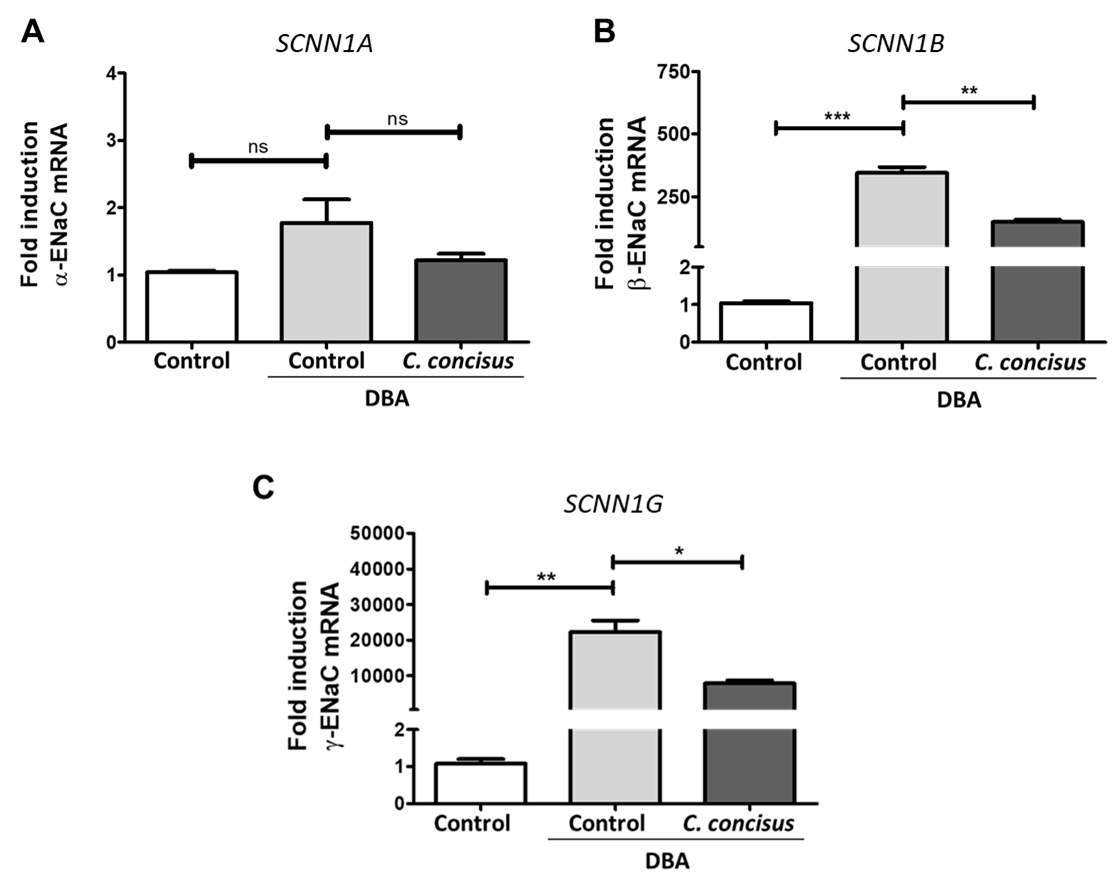


**Figure S1**. mRNA expression changes of ENaC subunits (-α, -β, -γ) in HT-29/B6-GR/MR cells 48 hours after *Camplyobacter concisus* infection. (A) mRNA expression of α-ENaC gene (*SCNN1A*) in controls, DBA-stimulated controls and *C. concisus*-infected cell monolayers (n = 4 each, ns = not significant, *p*>0.05). (B) mRNA expression of β-ENaC gene (*SCNN1B*) in controls, DBA-stimulated controls and *C. concisus*-infected cell monolayers (n = 4 each, ****p* < 0.01, ***p* < 0.01). (C) mRNA expression of γ-ENaC gene (*SCNN1G*) in controls, DBA-stimulated controls and *C. concisus*-infected cell monolayers (n = 4 each, ***p* < 0.01, **p* < 0.05). For statistical analysis, one-way ANOVA with Bonferroni’s Multiple Comparison Test was used and *p* < 0.05 was considered statistically significant. DBA = Dexamethasone, Butyrate, and Aldosterone.
