# Supplementary material for: Campylobacter concisus Impairs Sodium Absorption in Colonic Epithelium via ENaC Dysfunction and Claudin-8 Disruption
Source: Int J Mol Sci. 2020 Jan 7;21(2):373. doi: 10.3390/ijms21020373 (PMC7013563; doi:10.3390/ijms21020373)
Supplement: Supplementary file 1 [file ijms-21-00373-s001.zip › Supplementary Table S3.docx]

**Table S3. Upstream regulator analysis**

| Upstream regulator | Activation score | *p*-value of overlap | Target Molecule in dataset |
| --- | --- | --- | --- |
| INF-γ | 1,225 | 8,43E^-04^ | AQP1,ASNS,CEACAM5,CFTR,CHAC1,LAMP3,LCN2,  ***IL-32,*** SLC7A11,SLC7A5 |
| IL-1β | 0,340 | 4,39E^-03^ | CFTR,CRYAB,CYP1A1,FAM129A,***IL-32***,LCN2,SLC7A11 |
| Lipopolysaccharide (LPS) | 2,100 | 1,76E^-04^ | AQP1,CFTR,CRYAB,CYP1A1,***IL-32***,LAMP3,LCN2,LYZ,  S100A2,SLC7A11,TDO2,TRIB3,ULBP1 |
| IL-5 | 2,000 | 3,68E^-03^ | ASNS,HSPA6,PSAT1,SLC7A5 |
| NF-kB complex | 1,085 | 6,77E^-05^ | AQP1,CD7,CFTR,***IL-32***,LCN2,SLC7A5,TDO2,TRIB3 |
| Dexamethasone | 0,720 | 5,83E^-06^ | AQP1,ASNS,CEACAM5,CEACAM6,CEACAM7,CRYAB,  CYP1A1,DNER,FAM129A, ***IL-32****,* HSPA6,LAMA1,LCN2,SPINK13,TDO2,TRIM31 |

Upstream regulator analysis was performed with Ingenuity Pathway Analysis (IPA, Qiagen Silicon Valley, Redwood City, CA, USA) from RNA-Seq data to compare DBA-stimulated controls against *Campylobacter concisus* infection. The activation z-score, overlap *p*-value and target molecules of different upstream regulators activated by *C. concisus* 48 hours post-infection were shown in the table. The pathways for cytokines interferon-γ (INF-γ) and interleukin-1β (IL-1β), and bacterial LPS were activated and target IL-32 (referring to the scheme, Figure 11). Interleukin-5 (IL-5) was another cytokine found to be activated, but did not target IL-32. In addition, other upstream regulators, which were activated and target IL-32 were also displayed in the table (NF-kB as pro-inflammatory pathway or dexamethasone as anti-inflammatory pathway).
