# Supplementary figures and images for: Campylobacter concisus Impairs Sodium Absorption in Colonic Epithelium via ENaC Dysfunction and Claudin-8 Disruption
Source: Int J Mol Sci. 2020 Jan 7;21(2):373. doi: 10.3390/ijms21020373 (PMC7013563; doi:10.3390/ijms21020373)

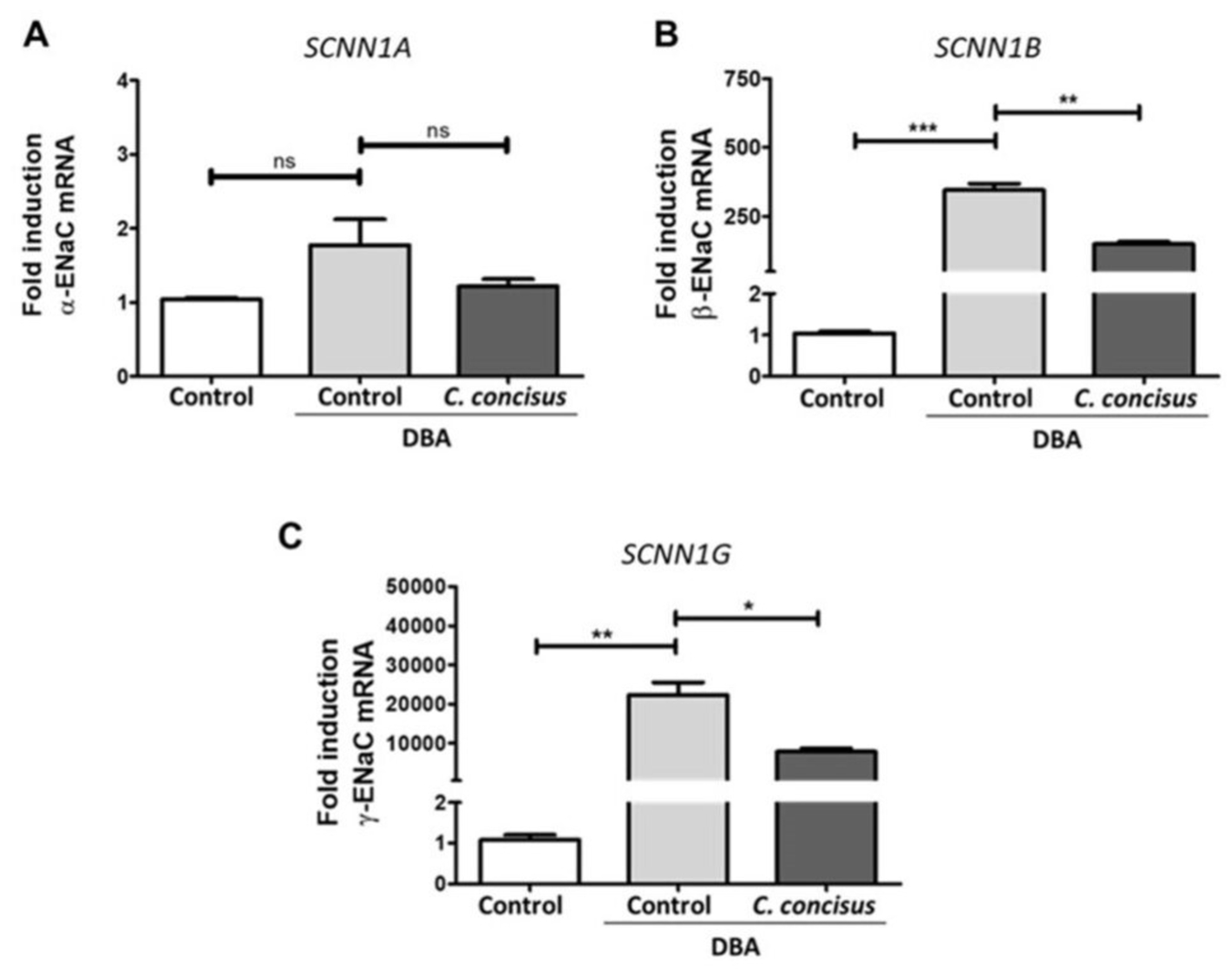

Supplement: Supplementary file 1 [file ijms-21-00373-s001.zip › Supplementary Figure S1.tif]
